# Analysis of malaria surveillance data in Ethiopia: what can be learned from the Integrated Disease Surveillance and Response System?

**DOI:** 10.1186/1475-2875-11-330

**Published:** 2012-09-17

**Authors:** Daddi Jima, Milliyon Wondabeku, Abebe Alemu, Admas Teferra, Nuraini Awel, Wakgari Deressa, Adamu Adissie, Zerihun Tadesse, Teshome Gebre, Aryc W Mosher, Frank O Richards, Patricia M Graves

**Affiliations:** 1 Public Health Emergency Management, Ethiopian Health and Nutrition Research Institute, Addis Ababa, Ethiopia; 2 WHO, Addis Ababa, Ethiopia; 3 Department of Epidemiology and Biostatistics, School of Public Health, Addis Ababa University, Addis Ababa, Ethiopia; 4 The Carter Center, Addis Ababa, Ethiopia; 5 The Carter Center, Atlanta, GA, USA; 6 Present address: International Trachoma Initiative Regional Office, Addis Ababa, Ethiopia; 7 Present address: School of Public Health, Tropical Medicine and Rehabilitation Sciences, James Cook University, PO Box 6811, Cairns, Qld, 4870, Australia

**Keywords:** Surveillance, Ethiopia, Malaria indicators, Incidence, Reporting completeness

## Abstract

**Background:**

Routine malaria surveillance data is useful for assessing incidence and trends over time, and in stratification for targeting of malaria control. The reporting completeness and potential bias of such data needs assessment.

**Methods:**

Data on 17 malaria indicators were extracted from the Integrated Disease Surveillance and Response System database for July 2004 to June 2009 (Ethiopian calendar reporting years 1997 to 2001). Reporting units were standardized over time with 2007 census populations. The data were analysed to show reporting completeness, variation in risk by reporting unit, and incidence trends for malaria indicators.

**Results:**

Reporting completeness, estimated as product of unit-month and health facility reporting, was over 80% until 2009, when it fell to 56% during a period of reorganization in the Ministry of Health. Nationally the average estimated annual incidence of reported total malaria for the calendar years 2005 to 2008 was 23.4 per 1000 persons, and of confirmed malaria was 7.6 per 1,000, with no clear decline in out-patient cases over the time period. Reported malaria in-patient admissions and deaths (averaging 6.4 per 10,000 and 2.3 per 100,000 per year respectively) declined threefold between 2005 and 2009, as did admissions and deaths reported as malaria with severe anaemia. Only 8 of 86 reporting units had average annual estimated incidence of confirmed malaria above 20 per 1,000 persons, while 26 units were consistently below five reported cases per 1,000 persons per year.

**Conclusion:**

The Integrated Disease Surveillance and Response System functioned well over the time period mid 2004 to the end of 2008. The data suggest that the scale up of interventions has had considerable impact on malaria in-patient cases and mortality, as reported from health centres and hospitals. These trends must be regarded as relative (over space and time) rather than absolute. The data can be used to stratify areas for improved targeting of control efforts to steadily reduce incidence. They also provide a baseline of incidence estimates against which to gauge future progress towards elimination. Inclusion of climate information over this time period and extension of the dataset to more years is needed to clarify the impact of control measures compared to natural cycles on malaria.

## Background

Malaria prevention and control interventions have recently undergone major scale-up in Africa, and malaria disease burden is reported to be declining in several countries [1], including Ethiopia and other East African countries [2-5]. However, there is complexity within countries, including large geographical variation in incidence and differing upward or downward trends between indicators, hospitals or areas [2,6,7]. Repeated representative nationwide malaria prevalence surveys are now becoming the norm, and there is a welcome emphasis on improving estimates of the impact of control measures on malaria mortality [8]. However, comprehensive longitudinal data sources measuring several malaria indicators monthly or weekly at multiple sites are relatively few, and more are needed [9,10].

While most countries have routine morbidity and mortality reporting systems, distrust of their quality for malaria surveillance is widespread [11,12] and may sometimes be justified. However, there are many examples of carefully assessed routine malaria surveillance data making essential contributions to understanding the malaria burden, how it varies over space and time, and the impact of control measures and climate on malaria [5,13-20]. A more frequent problem is the collection of large amounts of data that are never collated or used, with consequent degrading of reporting as health workers receive no feedback for their surveillance effort. There is little alternative to routine surveillance for understanding inter-annual and seasonal trends over sub-national areas, and since there are often multiple sources, triangulation between them can assess which ones are providing high quality data as well as lend reassurance that observed trends are real.

Recognition of the importance of improvement of quality of routine surveillance for infectious diseases [21] led to major input by WHO and CDC into strengthening and streamlining of this effort through the Integrated Disease Surveillance and Response (IDSR) system [22] starting in the 1990s. The IDSR matrix included originally 19 diseases (pandemic influenza being later added) in three categories: 1) major endemic diseases of public health importance; 2) diseases targeted for eradication and elimination; and 3) epidemic prone diseases. Malaria comes under the first category in many countries but may be in the third category in less endemic countries like Ethiopia and Eritrea.

Some important features of IDSR and its training materials were the use of standard case definitions, the establishment of action thresholds for epidemics, the use of information in response, provision of feedback, and continuous evaluation [22]. Evaluations of IDSR systems have occurred [21,23], but there has been no detailed use and evaluation of IDSR for monitoring trends in malaria in Ethiopia.

The total estimated population of Ethiopia in 2007 was 73,918,505 [24]. Administrative levels of Ethiopia consist of regional states usually divided into zones, which have populations (in 2007) averaging 785,000 and ranging from 35,000 to 2,970,000. There are some ‘special districts’, administratively equivalent to zones. The next levels are *woredas* (districts) with populations averaging 140,000 and ranging from 8,000 to 340,000, followed by *kebeles* (the lowest government administrative unit, divided into villages or ‘development teams’). *Kebeles* have at least 900 families or about 4,500 to 5,000 persons. The number of hospitals per region varies, with at least one in each region. Zones may or may not have a hospital, but have at least one health centre. Each *woreda* (district) usually has one or more health centres and a number of health posts or stations in more rural areas. Starting in 2005, the health station category was phased out and each *kebele* is now intended to have at least one health post staffed by two Health Extension Workers, reporting to the cluster health centre. The national health management information system has recently undergone major reorganization. Each health facility reports quarterly on morbidity, mortality, and health resource and preventive indicators through that system.

Ethiopia implemented Integrated Disease Surveillance (IDSR) reporting from all hospitals and health centres using a one page form (see Additional file 1). Most diseases were reported on the monthly form, but certain high priority indicators were to be reported immediately. Health stations and health posts were not formally included in the initial phase. While IDSR data exist on paper at the district level, they were available in electronic form at zone and Regional Health Bureau level in an EpiInfo database entered in Addis Ababa, formerly under the Disease Prevention and Control Department, but now at the Public Health Emergency Management (PHEM) center at Ethiopian Health and Nutrition Research Institute.

This paper presents and assesses national surveillance malaria data from the IDSR system at the zone (reporting unit) level in Ethiopia over a five-year period July 2004-June 2009. The goals are 1) to investigate the completeness of reporting for malaria indicators in the IDSR system, 2) to stratify malaria risk by area of the country (zone); 3) to contribute to the information on whether and how the malaria burden in the country is changing over the time period, which coincided with the time of major scale up of malaria control efforts in the country. After this time period (in 2009), the reporting format changed, health posts were included in reporting, and the time interval for reporting changed to weekly during major Ministry of Health reorganization.

## Methods

### Malaria indicators in IDSR

The IDSR reporting form (see Additional file 1) includes 17 malaria items encompassing total malaria cases (clinical and confirmed) for out-patients, in-patients and deaths; confirmed out-patient malaria cases by species; in-patient cases and deaths for malaria with severe anaemia; and out-patients, in-patients and deaths for malaria in pregnancy. All are given by age group <5 years and > = 5 years except malaria in pregnancy indicators which only apply to women of childbearing age.

The Epi Info (Windows) IDSR database was imported into Access 2003 and expanded to include all eligible zone-months. Several variables were renamed for clarity and some summary malaria indicators were created in Access 2007, such as combining different age groups and estimating total confirmed malaria by summing cases of both species (see Additional file 2).

Incidence estimates for summary malaria indicators by year and month were obtained by summing the appropriate indicator over the time period in question using the relevant population denominator for each reporting unit (see Additional file 3).

### Reporting time periods sites, units and population denominators

The Ethiopian Calendar (EC) is used in the country, rather than the Gregorian (Western) calendar (GC). There are 13 months in the EC, 12 of 30 days and a short remaining month. The EC year runs from the equivalent of GC September to August, with a 7 to 8 year lag period behind the GC, whereas the reporting year for the Ministry of Health is GC July to June. The time period of this report is July 2004 to June 2009, which with the lag period is equivalent to EC reporting years 1997 to 2001.

In 2004 (EC 1997), there were 87 original IDS reporting units entered in the database at national level. Reporting units are mostly zones, but also include special *woredas* (districts), sub-cities, towns and referral hospitals that report separately. For convenience, these are referred to as ‘reporting units’ or just ‘units’ rather than zones. The number of reporting units had gradually increased to 108 by 2008–2009 (EC 2001) by the splitting of zones and the addition of referral hospitals and towns, and the strategy to adjust for this change is described below. In July 2007, five referral hospitals in Addis Ababa were added, five zone splits occurred, and 10 towns were split from their local zones and added as separate reporting units. One more referral hospital in Addis was added in July 2008. One zone in Somali region started reporting in July 2005 (one year late). Zones in Somali region that stopped reporting (9 in number) after June or December 2007 have been excluded from the count of ‘eligible months’ (reporting denominator) for the appropriate periods.

Reporting completeness was assessed based on the information available in the 108 units, after allocating eligible reporting time periods to each unit. Each monthly report showed a number of sites ‘expected to report’ which was taken as the number of eligible sites, totaled over each reporting unit.

For assessing malaria incidence, the 108 units reported by 2009 were collapsed into the original 87 units of 2004 to ensure consistent population denominators, estimated from the 2007 census. One of the original 87 units (Gambella Special Woreda) did not have a separately reported population so was combined with its surrounding area, giving 86 units. Recently added referral hospitals and newly demarcated towns were assigned to their original local zone for malaria incidence estimates. Split zones were reunited to their original zone as in 2004 (EC 1997) (see Additional file 3).

Most data were obtained in digital form from the database in the Public Health Emergency Management (PHEM) center, Addis Ababa; missing month reports from Amhara region that had never been submitted or entered were obtained by visiting the Regional Health Bureau in Bahir Dar in early 2010.

### Data quality

Factors affecting quality of IDSR data for reflecting disease trends include accuracy of the diagnosis according to the case definitions, accuracy of data recording and entry, completion and correct summary of the monthly forms at all levels, submission of the forms to next higher level, timeliness of reporting, and consistency in reporting over time.

While case definitions for the reporting form were clear and had been the subject of training, diagnosis is done by hundreds of staff members at the health centers and hospitals and its accuracy therefore cannot be assessed here. If accuracy of diagnosis remains relatively constant over time, it should not introduce bias. Occasional outliers or missing items within a unit-month due to either reporting or data entry errors (unresolvable at this time) were observed. Clear outliers or inconsistencies such as the number of confirmed cases of a disease being greater than the total number of cases, or cases of one species exceeding the total number of cases of both species were re-coded to ‘missing’. If reports existed but were not submitted from *woreda* to zone level, or received but never entered, this was reflected in the database as a completely missing unit-month report.

### Data timeliness, completeness and consistency

While timeliness of reports is vital for detecting and responding to epidemics, it is not evaluated here since the focus is on completeness of reporting to observe relative incidence between zones as well as trends over a five-year period. The numbers of sites (health facilities) reporting on time and those reporting late were therefore combined to arrive at an overall proportion of sites reporting for each unit-month.

The first focus of this evaluation is reporting completeness and consistency based on the expected number of eligible sites, reporting units and months. Increase in number of both eligible sites and reporting units (due to reconfiguration of boundaries) affected reporting consistency. One would expect a slow increase in the number of eligible sites as new health centres and hospitals were built (and this was observed in increase in number of eligible sites), but since the population served remains relatively constant, this increase in sites would not necessarily bias the disease trend unless access to care increased greatly. Boundary reconfiguration was addressed in the compilation of the incidence data by re-combining newly defined areas (e.g. split zones or separate reporting from towns) with their previous reporting units for assessing disease trends, depending on availability of population denominators. Thus 108 reporting units were compressed into 86. Change in reporting completeness over time is the factor most likely to bias disease trends.

Since the database initially did not contain any zone-months lacking reports, a full list of eligible months for each year and reporting unit was first generated. The completeness of reporting was calculated as the product of the following two proportions:

1) **percentage of unit-month reports:** For each zone (reporting unit), the number of months with any report divided by the eligible months.

2) **percentage of site reports:** For each unit-month: the proportion of reports from eligible sites (health centres or hospitals) that were received at the reporting unit level and included in their reports.

## Results

### Reporting completeness

Reporting was assessed after adjustment for the time each unit was supposed to be reporting (‘eligible months’). Details of the reporting units and eligible months are given in Additional files (see Additional files 4 and 5). Overall there were 5,508 unit-months eligible for reports.

**Figure 1:**
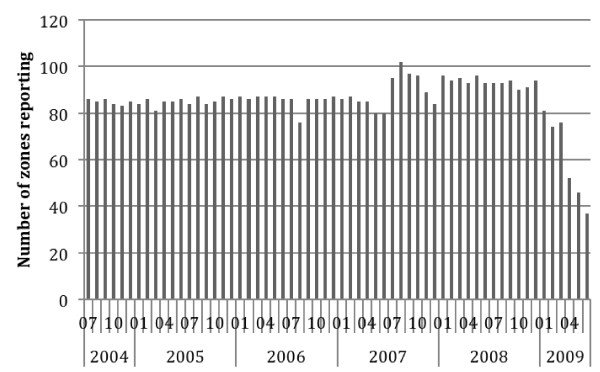
Number of zone units reporting by month, Ethiopia, July 2004 to Jun 2009.

The number of units actually reporting each month is shown in Figure 1. An increase in July 2007 occurred, when many new reporting units were added. There was a progressive drop off in reporting starting in January 2009 during Ministry of Health reorganization and planning for conversion to a weekly system.

Within each reporting unit, there was a variable number of reporting sites (health centres and hospitals) ranging from 1 to 51. The total number of eligible sites per month increased from 818 in January 2005 to 1258 in December 2008 as new facilities were built; it averaged 844 per month over the five year period. The reported number of eligible sites fluctuated unexpectedly in some zones, suggesting that the denominator of reporting sites was not always reported correctly. However, both the number of eligible sites and the number of sites reporting increased overall with similar time trends, and the proportion of sites reporting was equal to or less than the number of eligible sites in all but one case.

Overall the proportion of eligible unit-months for which reports were received was 92.8%. There were 392 missing month reports (7.1%) out of a potential 5,508. The percent of sites reporting was 88.0% in available unit-month reports (44,607 sites stated to have reported out of 50,667 eligible). Multiplying unit-month and site reporting percentages gave an overall estimate of 81.7% reporting completeness (Table 1).

**Table 1:** Reporting completeness for zones (unit-months), sites and overall by region

**Region**	**Eligible Unit-months**	**Unit-months with Reports**	**% Unit- months with Reports**	**Eligible Sites**	**Sites with Reports**	**% Sites with Reports**	**Overall % Reporting**
							(Unit-months * Sites)
Addis Ababa	738	720	97.6	8776	8059	91.8	89.6
Afar	300	285	95.0	1111	988	88.9	84.5
Amhara	660	570	86.4	6808	6019	88.4	76.4
Benishangul Gumuz	300	259	86.3	3133	2538	81.0	69.9
Dire Dawa	60	60	100.0	673	611	90.8	90.8
Gambella	264	205	77.7	641	478	74.6	57.9
Harari	60	54	90.0	384	312	81.3	73.1
Oromia	1128	1047	92.8	13302	11288	84.9	78.8
SNNPR	1284	1239	96.5	10529	9833	93.4	90.1
Somali	354	316	89.3	641	361	56.3	50.3
Tigray	360	355	98.6	4669	4120	88.2	87.0
TOTAL	5508	5110	92.8	50667	44607	88.0	81.7

Note: “unit-months*sites” is the product of proportion of zones (reporting units) and proportion of sites that reported.

By region, reporting completeness ranged from 50.3% in Somali region to 90.8% in Dire Dawa (Table 1). By reporting unit, reporting completeness varied from 34.3% in Korahe zone in Somali region to 100% for two referral hospitals in Addis Ababa (see Additional file 5).

The percent of months for which there were reports dropped from 98.4% in 2004–2005 to 78.3% in 2008–2009, mainly due to the drop off in early 2009 (Table 2). Until Jan 2009, over 90% of units were reporting each month. The percent of sites reporting within units did not decline over time, ranging from a low of 86.3% in Jan to Jun 2005 to 89.8% in Jan to Jun 2009 (Table 2), suggesting that where the system was still in operation sites were still reporting, but unit months were not being compiled or forwarded. Overall reporting rate (unit-months multiplied by sites) was over 80% for each six month period up to the end of 2008.

**Table 2:** Reporting completeness for zones (reporting units), sites and overall by six-month period

**Year EC**	**Year GC**	**Six- month period**	**Eligible Unit-months**	**Unit-months with Reports**	**% Unit- months with Reports**	**Eligible Sites**	**Sites with Reports**	**% Sites with Reports**	**Overall % Reporting**
									(Unit-months * Sites)
1997	2004	Jul-Dec	516	509	98.6	NA	NA	NA	NA
1997	2005	Jan-Jun	516	507	98.3	4624	3991	86.3	84.8
1998	2005	Jul-Dec	522	513	98.3	4774	4166	87.3	85.8
1998	2006	Jan-Jun	522	520	99.6	5243	4602	87.8	87.4
1999	2006	Jul-Dec	522	507	97.1	5361	4702	87.7	85.2
1999	2007	Jan-Jun	522	503	96.4	5567	4928	88.5	85.3
2000	2007	Jul-Dec	624	563	90.2	6181	5482	88.7	80.0
2000	2008	Jan-Jun	588	567	96.4	6697	5916	88.3	85.2
2001	2008	Jul-Dec	588	555	94.4	7033	6177	87.8	82.9
2001	2009	Jan-Jun	588	366	62.2	5152	4627	89.8	55.9
TOTAL			5508	5110	92.8	50632	44591	88.1	81.7

Note: “Unit-months*sites” is the product of proportion of zones (reporting units) and proportion of sites that reported.

Examining reporting rates by region over time shows some important differences between them (Table 3). Most regions show the drop off in submitting monthly reports in early 2009, but it was more severe in some regions (Amhara and Harari for example) than others (Oromia and SNNPR), and the decline started earlier in 2008 in some cases (Afar, Benishangul Gumuz and Gambella). There were also low reporting rates in Amhara and Gambella in late 2007.

**Table 3:** Reporting completeness by region and six month period

	Overall % of Reports Received (Unit-month * Site)										
**Year EC**	**1997**	**1997**	**1998**	**1998**	**1999**	**1999**	**2000**	**2000**	**2001**	**2001**	**TOTAL**
**Year GC**	**2004**	**2005**	**2005**	**2006**	**2006**	**2007**	**2007**	**2008**	**2008**	**2009**	
**Six-month period**	**Jul-Dec**^1^	**Jan-Jun**	**Jul-Dec**	**Jan-Jun**	**Jul-Dec**	**Jan-Jun**	**Jul-Dec**	**Jan-Jun**	**Jul-Dec**	**Jan-Jun**	
Addis Ababa	(100)	88.1	92.5	91.9	93.1	89.1	92	90.1	92.4	77.8	89.6
Afar	(100)	94.4	97.3	99.1	99.2	94.7	85.2	97.6	70.1	38.4	84.5
Amhara	(100)	91.7	86	89.8	89.4	86.1	58.6	88.6	78.3	10.1	76.4
Benishangul Gumuz	(96.7)	79.6	87.9	83.8	88.1	72.5	73	66.8	38	31.9	69.9
Dire Dawa	(100)	90.5	100	100	100	100	100	96.7	76.7	69.1	90.8
Gambella	(100)	69.2	71.3	57.7	70.3	71	45.9	68.3	38.6	30	57.9
Harari	(100)	66.7	81.3	83.3	79.2	85.4	85.4	83.3	85.4	0	73.1
Oromia	(100)	77.3	75.6	83	76.8	78.4	82.1	86.3	89.1	58	78.8
SNNPR	(100)	92.6	90.7	94.2	92.9	94	91.2	90.3	92	69.9	90.1
Somali	(95.8)	61.2	72.2	51.1	41.9	57.8	19	NA	NA	NA	50.3
Tigray	(88.9)	100	98.5	95.6	93.4	91.3	88.5	74.8	85.3	88.7	87.0

^1^ Reporting rate for unit-month only; site numbers were not required to be reported until start of 2005.

Note: “Unit- month*Site” is the product of proportion of zones (reporting units) and proportion of sites that reported.

### National level malaria indicators

**Figure 2:**
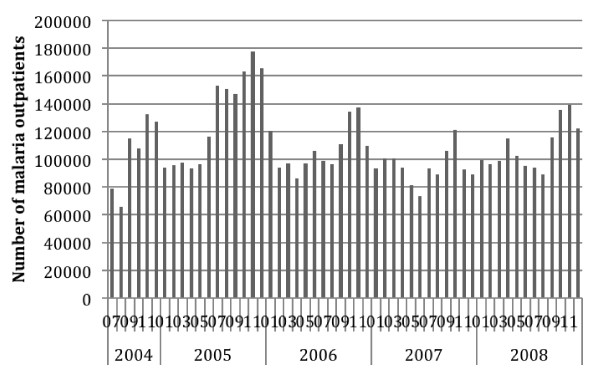
Reported Total Malaria Out-patient Cases at Health Centres and Hospitals, Ethiopia, July 2004 to December 2008.

**Figure 3:**
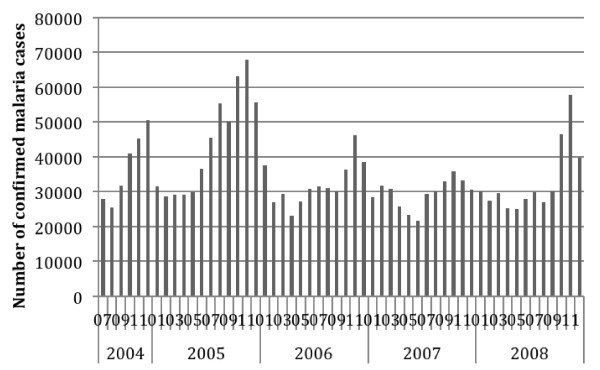
**Reported Confirmed Malaria Out-patient Cases at Health Centres and Hospitals, Ethiopia, July 2004 to December 2008.***P. falciparum, P. vivax.*

Table 4 shows the summary national level malaria indicators by six month period. Incidence is by six month period and per 1000 persons for out-patient indicators, per 10,000 for in-patients and per 100,000 for mortality. Taking into account the drop off in reporting in early 2009, the malaria cases data for Jan- June 2009 are not regarded as being representative or valid. Therefore, malaria indicators by month are reported only up to the end of 2008 in the figures below, including Figure 2 (Total Out-patient Cases) and Figure 3 (Confirmed Cases). Annual incidence is reported by calendar year (GC) for 2005 to 2008 in Table 5.

**Table 4:** Reported national incidence for malaria indicators from IDSR surveillance data, by six month intervals

**Year EC**	**1997**	**1997**	**1998**	**1998**	**1999**	**1999**	**2000**	**2000**	**2001**	**2001**	**Average**	
**Year GC**	**2004**	**2005**	**2005**	**2006**	**2006**	**2007**	**2007**	**2008**	**2008**	**2009**		
**Six-month period**	**Jul-Dec**	**Jan-Jun**	**Jul-Dec**	**Jan-Jun**	**Jul-Dec**	**Jan-Jun**	**Jul-Dec**	**Jan-Jun**	**Jul-Dec**	**Jan-Jun**	**Jul-Dec**	**Jan-Jun**^1^
Number of reporting units	85	85	86	86	86	86	84	77	77	*77*	83.6	84.3
Overall % reporting completeness	NA	84.8	85.8	87.4	85.2	85.3	90	85.2	82.9	*55.9*	-	-
Total out-patient malaria cases /1,000	11.04	10.30	16.87	10.57	11.83	8.98	10.06	10.92	13.03	*7.65*	12.57	11.36
Malaria in-patients /10,000	4.36	4.16	6.54	3.76	2.59	2.03	1.86	2.12	2.15	*1.54*	3.50	3.29
Malaria in-patient deaths /100,000	1.97	1.71	2.31	1.18	1.07	0.71	0.68	0.73	0.64	*0.54*	1.33	1.20
*Pf* cases /1,000	2.63	2.18	4.34	1.96	2.47	1.74	2.14	1.83	2.79	*1.86*	2.88	2.38
*Pv* cases /1,000	1.40	1.02	1.76	1.10	1.45	1.05	1.26	1.23	1.80	*1.25*	1.53	1.27
Confirmed malaria cases /1,000	4.03	3.20	6.10	3.06	3.92	2.79	3.40	3.06	4.59	*3.10*	4.41	3.65
Malaria with severe anaemia in-patients /10,000	0.40	0.39	0.73	0.25	0.30	0.26	0.25	0.28	0.40	*0.13*	0.41	0.35
Malaria with severe anaemia deaths /100,000	0.30	0.29	0.44	0.18	0.12	0.07	0.08	0.17	0.19	*0.07*	0.23	0.19
Malaria in pregnancy out-patients/1000	0.15	0.14	0.20	0.16	0.16	0.13	0.13	0.16	0.16	*0.13*	0.16	0.15
Malaria in pregnancy inpatients /10,000	0.10	0.09	0.18	0.09	0.08	0.06	0.05	0.08	0.08	*0.05*	0.10	0.09

^1^ (excl 2001 EC/2009 GC).

**Table 5:** Reported national annual incidence for malaria indicators from IDSR surveillance data, GC calendar year

**Year (GC**)	**2005**	**2006**	2007*	2008*	**Average**
Total out-patient malaria cases/1000	27.098	22.400	19.928	23.950	23.388
Malaria in-patients/10,000	10.670	6.343	3.976	4.270	6.383
Malaria in-patient deaths/100,000	4.010	2.246	1.401	1.365	2.282
*Pf* cases/1,000	6.510	4.430	4.061	4.622	4.923
*Pv* cases/ 1,000	2.768	2.549	2.426	3.025	2.691
Confirmed malaria cases/1,000	9.278	6.979	6.487	7.647	7.614
Malaria with severe anaemia in-patients/10,000	1.114	0.541	0.533	0.678	0.720
Malaria with severe anaemia deaths /100,000	0.729	0.298	0.146	0.360	0.387
Malaria in pregnancy out-patients/1,000	0.345	0.309	0.260	0.319	0.309
Malaria in pregnancy in-patients/10,000	0.264	0.169	0.115	0.161	0.179
Malaria in pregnancy deaths/100,000	0.096	0.057	0.032	0.035	0.056

*Excludes Somali Region zones that reported only up to part way through 2007.

**Figure 4:**
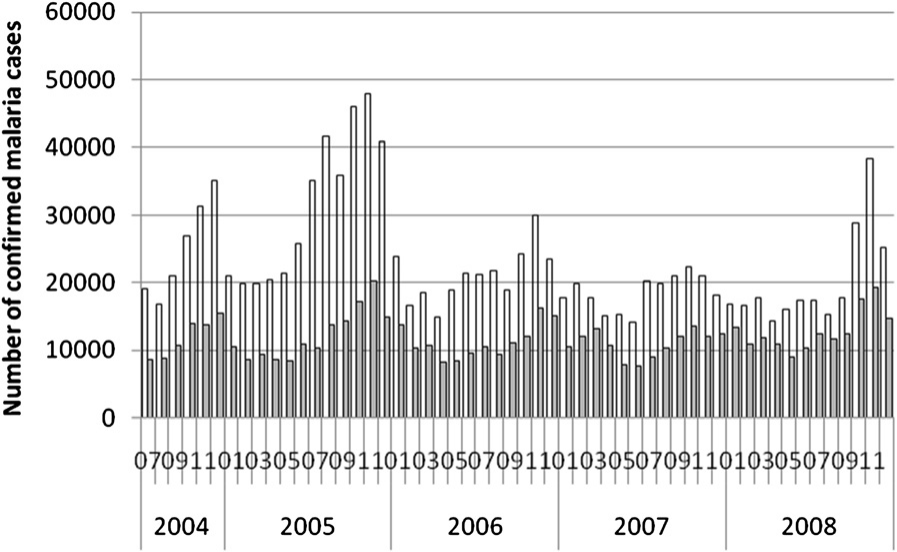
Reported Malaria In-patients at Health Centres and Hospitals, Ethiopia, July 2004 to December 2008.

**Figure 5:**
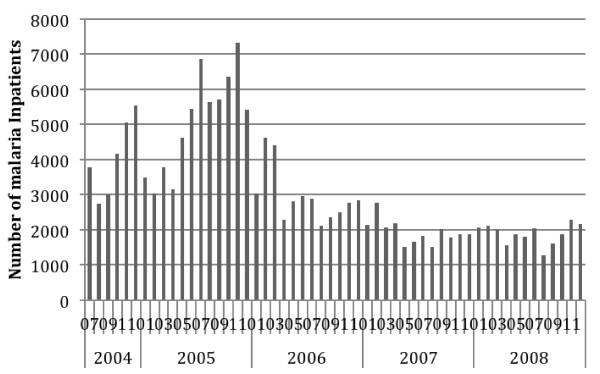
**Reported Malaria In-patient Deaths at Health Centres and Hospitals, Ethiopia, July 2004 to December 2008.** Open bars = Malaria deaths; Filled bars = Malaria in pregnancy deaths.

**Figure 6:**
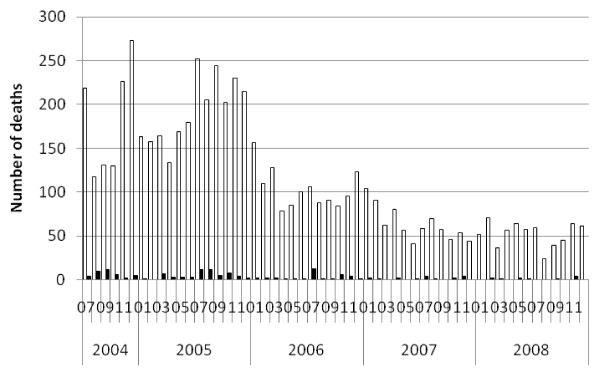
**Reported Malaria with Severe Anaemia In-patients and Deaths at Health Centres and Hospitals, Ethiopia, July 2004 to December 2008.** Open bars = in-patients with malaria and severe anaemia; Filled bars = deaths with malaria and severe anaemia.

**Figure 7:**
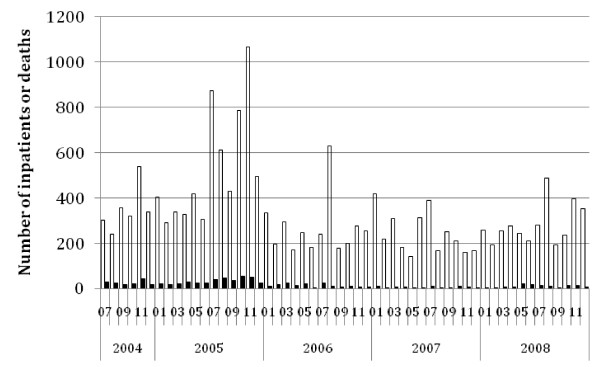
**Reported Confirmed Malaria Out-patient Cases by Species at Health Centres and Hospitals, Ethiopia, July 2004 to December 2008.** Open bars = *P. falciparum*; Filled bars = *P. vivax*.

Nationally the average estimated annual incidence of reported total malaria in the overall population was 23.4 per 1000 persons and of confirmed malaria was 7.6 per 1,000 per year over the four calendar years 2005 to 2008 (Table 5). Thus total cases were about three times higher than confirmed cases. Reported malaria in-patient admissions and deaths averaged 6.4/10,000 and 2.3 per 100,000 per year respectively. Reported malaria in-patient admissions and deaths, including deaths from malaria with severe anaemia, declined two- to threefold during this period (Table 5, Figures 4, 5 and 6). However, there was no clear declining trend in the number of malaria out-patients, either total malaria or confirmed malaria (Figures 2 and 3) over this time period. There did appear to be increasing relative numbers of *Plasmodium vivax* among confirmed malaria cases (Figure 7).

Relatively few malaria deaths are reported, so the number of in-patients is a more robust and precise indicator of severe cases than mortality. As a further indicator of severe malaria and mortality potentially caused by malaria, the IDSR system also reported the number of in-patients and deaths with malaria and severe anaemia, which also demonstrated declining trends particularly in the first two years studied (Table 5 and Figure 5). Few malaria in pregnancy cases, admissions or deaths were reported making these less useful indicators than total and confirmed malaria out-patient cases and admissions.

### Zone (reporting unit) level malaria indicators

**Figure 8:**
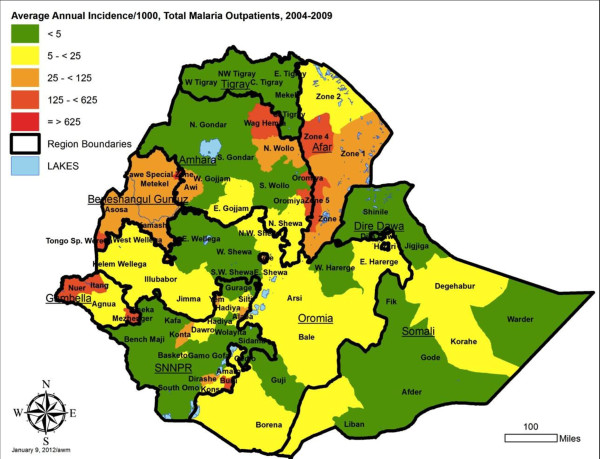
Average annual incidence of total reported malaria out-patients per 1000 population, by reporting unit, Ethiopia, July 2004 to June 2009.

**Figure 9:**
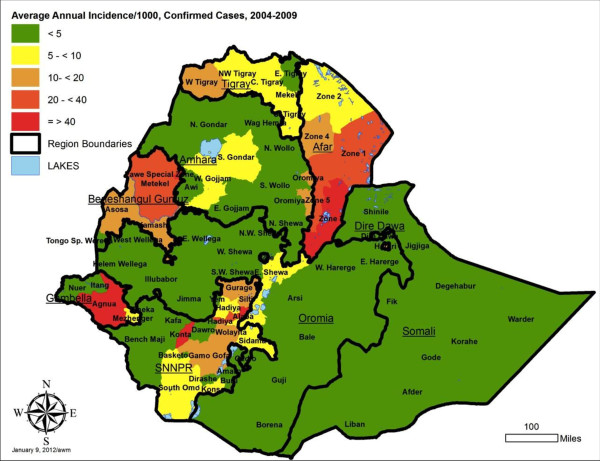
Average annual incidence of reported confirmed malaria cases per 1000 population, by reporting unit, Ethiopia, July 2004 to June 2009.

To contribute to geographical stratification of malaria risk in Ethiopia, estimates were also derived for annual incidence per 1000 persons by reporting unit, for both total malaria and confirmed malaria out-patient cases. The average annual incidence over the five year period is shown, stratified by five levels, in Figures 8 and 9, with data by reporting unit given in Additional files (see Additional files 6 and 7). Note that Somali region zone data are missing for 2007–2008 and 2008–2009, so the average incidence is estimated based on the earlier three years for these zones. Also, the under-reporting in many zones in late 2009 biases the annual incidence estimates for 2008–2009 downwards.

**Figure 10:**
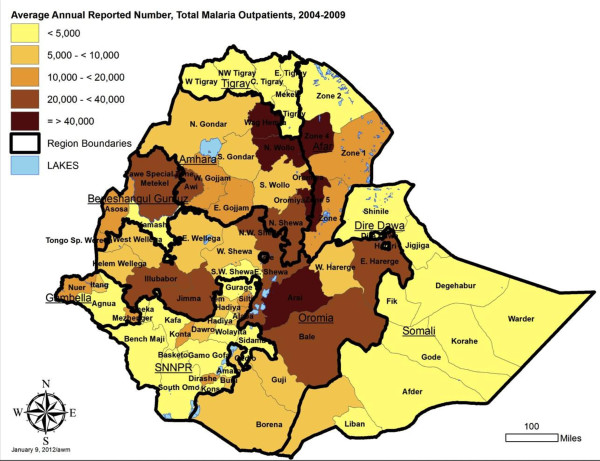
Average annual number of total reported malaria out-patients by reporting unit, Ethiopia, July 2004 to June 2009.

**Figure 11:**
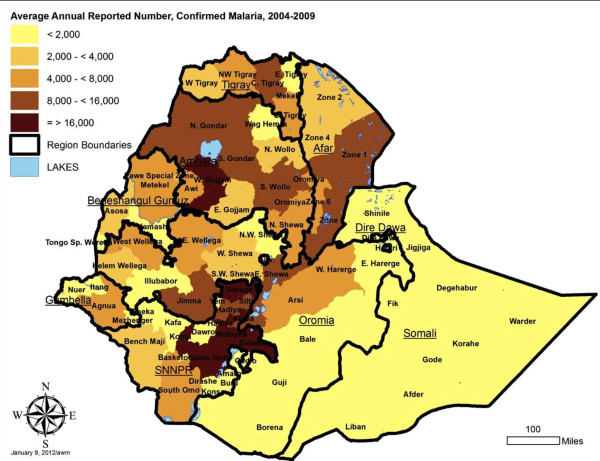
Average annual number of reported confirmed malaria cases by reporting unit, Ethiopia, July 2004 to June 2009.

Due to variation in size of zone populations, the highest expected annual number of cases is not necessarily in zones with the highest incidence. Therefore, in addition to incidence, maps are presented showing the estimate of actual number of cases reported annually. Figure 10 shows estimated total malaria cases, and Figure 11 confirmed cases.

By reporting unit, the annual incidence of reported total malaria (clinical plus confirmed - Figure 8) in out-patients ranged from an average of 0.3 cases per 1,000 per year in Addis Ababa - Bole subcity to 535.2 per 1000 per year in Benishangul Gumuz – Pawe special woreda [see Additional file 6]. Average reported confirmed malaria incidence per 1000 per year (Figure 9) ranged from 0.2/1000 per year in Addis Ababa –Bole subcity to 73.2/1000/year in SNNPR – Alaba special woreda [see Additional file 7].

The reporting units predicted to have the highest number of malaria out-patient cases (>50,000) in an ‘average’ year are zones 4 and 5 in Afar region; North Wello, Oromia and Waghimra zones in Amhara region; Pawe special woreda in Benishangul Gumuz region; and Arsi zone (combined Arsi and West Arsi) in Oromia region (Figure 10; [see Additional file 6]). The units predicted to have the highest number (>15,000) of confirmed malaria cases in an ‘average’ year were all in SNNPR region: Alaba, Gamo Gofa, Guraghe, Kembata-Tembaro, Sidama, and Wolayta (Figure 11 [see Additional file 7]).

## Discussion

This study carefully examined the completeness of reporting of malaria indicators for the IDSR system in Ethiopia between 2004 and 2009, and concluded that at over 80% it was of sufficient quality to provide estimates of malaria incidence by reporting unit (usually zone) and month until the end of 2008. The results suggest marked decline in numbers of malaria and malaria-related in-patients and deaths over the period, perhaps as a result of scale up of interventions in 2007, although little drop in out-patient case numbers was observed.

It must be remembered that the IDSR system in 2004–2009 expected reports from health centers and hospitals only. During the last few years, there has been a large expansion in the number of health posts in the country [25], which diagnose and treat an increasing proportion of out-patient malaria cases. This could bias the number of out-patient cases seen at higher-level facilities downwards, although this factor may be counteracted if health post cases are reported through their supervisory health centre. However, health posts do not accept in-patients. It is possible that expansion of access to diagnosis and treatment through Health Extension Workers contributed to earlier and more effective care-seeking and effective treatment, with consequent reduced incidence of severe malaria cases and mortality at health centers and hospitals. Since 2004, reports of outbreaks or epidemics due to malaria in the study zones of the country have also been very low.

A limitation of the data set was that it was not possible to independently verify the accuracy of the reported number of eligible sites in each reporting unit, and under-reporting of the number of such sites would overestimate the completeness of eligible site reporting. However, examination of time trends did not indicate any significant changes over time that would bias the results.

Incidence of total malaria out-patients (clinical and confirmed malaria) in the overall population averaged 23.4 per 1,000 persons per year over 2005 to 2008, and was about three fold higher than incidence of confirmed malaria at 7.6 per 1,000 per year. Both indicators should be taken into account in stratification of risk between zones (reporting units), since the true incidence probably lies between the two. Differences in ranking of units by the level of risk observed and by predicted absolute number of total and confirmed malaria cases (Figures 8, 9, 10, and 11) emphasize the need to consider more than one malaria indicator (including in-patient admissions) in stratification schemes and prediction of needs for prevention interventions and drugs. It would also help to assess effectiveness of malaria control if intervention planning was conducted in the same geographical units as the incidence estimates.

A separate source of malaria incidence estimates over the period of this study is the Health Management Information System (HMIS), Federal Democratic Republic of Ethiopia, in which the estimated annual incidence of reported malaria for the whole country was 107 per 1,000 in 2004 and 55 per 1,000 in 2009. Severe malaria cases reported through the HMIS declined from 148 per 100,000 in 2004 to 54 per 100,000 in 2009. Thus a steeper decline in severe cases than out-patient cases was also observed in the national statistics reported through a different system, although incidences of overall reported cases and of severe cases were higher in the national HMIS data than in the IDSR reports. This is expected given that IDSR data compiled here are only from health centres and hospitals rather than all health facilities, and thus do not capture the cases treated at the large network of health posts and also private facilities. The importance of the dataset here is to show trends and comparisons between zones/reporting units over time and to stratify by reporting unit.

The IDSR dataset is a rich source of information for stratification of malaria risk by zone in Ethiopia, using reported empirical data rather than climate predictions. It is known that not all of Ethiopia is at malaria risk since some areas are too high and others too dry for transmission [26]. However, it was not possible to adjust the incidence estimates for reporting units by a standard factor of population at malaria risk because the proportion of each zone or reporting unit at risk was highly variable. The overall incidence by reporting unit is affected both by the proportion of zone at risk and the intensity of transmission in the area at risk. Incidence defined by sub-zonal units such as *woredas* would be highly valuable in future.

While the pattern of incidence stratification shown by these empirical data fits reasonably well with maps predicted from climate data [27-29], reviewed in [30], the data suggest that some areas of the country such as Afar and Beneshangul Gumuz regions have higher incidence than generally realized and deserve increased control programme attention. In arid regions where incidence is higher than expected, irrigation projects may be influencing malaria transmission.

It is also important to note that patients do not always seek treatment in their zone of residence. In most zones, boundary crossing may occur in both directions and hence is unlikely to introduce systematic bias, but it may inflate estimates in urban areas. For example, those living in the surrounding areas of towns like Harar or Dire Dawa may cross the reporting unit boundary and inflate the incidence estimates in such towns. While this may overestimate the risk of malaria within the town, it is nevertheless a useful estimate of need for services in those places. Without detailed studies of patients’ residence, this must be accepted as a limitation of the data.

Prediction of the absolute numbers of cases expected, generated here by reporting unit from the IDSR data, confirms the important contribution of highly populated zones such as those in Eastern Amhara Regional State and the rift valley of Oromia and SNNPR to the large numbers of cases reported in Ethiopia annually [26]. These large regions and zones tend to be the ones already given the most attention by the malaria control programme, but the current study may assist in refining the programme’s efforts. Obviously it would be ideal to generate risk maps by *woreda* rather than zone, but even at zone level clear seasonal and inter-annual patterns are apparent and can be used to establish thresholds for defining future epidemics. In general, both absolute case numbers and the use of incidence when comparing between zones and regions will improve the ability of Regional Health Bureaus and the FMOH to plan resources appropriately, improve targeting of malaria control efforts, and allow better evaluation of the programme.

Since these data were reported, a new weekly system has been instituted, in which the malaria indicators have been condensed and reconciled with HMIS indicators to reduce workload for health staff in filling multiple forms. All health facilities down to health posts are now required to report. The number of persons tested for malaria confirmation (by slide or rapid test) has been included as well as total malaria and confirmed malaria cases to provide a denominator for test positivity rate. The change to the new system occurred during extensive business process restructuring in the Ministry of Health and was not implemented across all the states at once.

It remains to be seen whether these low rates in many zones and for the country overall remained at such levels in 2010 and 2011. Inclusion of climate information over this time period [31] and extension of the dataset to more years is needed to clarify the role of control measures compared to natural cycles. It is possible that the declines seen merely represent the down side of an epidemic cycle, but it may be real and sustainable, due to improved health system delivery, better malaria prevention (LLIN and IRS) and use of effective drugs. Given that Ethiopia is considering malaria elimination [32], it is useful to note that between 2004 and 2009, only 8 reporting units had average annual estimated incidence of confirmed malaria (reported to IDSR) above 20 per 1,000 persons, while 26 units (excluding Somali region) were consistently below five reported cases per 1,000 persons per year. Elimination at least from selected zones thus appears a possibility while attention is given to reducing incidence further in the other zones.

## Conclusion

The Integrated Disease Surveillance and Response System functioned well over the time period mid 2004 to the end of 2008. The data suggest that the scale up of interventions has had considerable impact on malaria in-patient cases and mortality, as reported from health centres and hospitals. These trends must be regarded as relative (over space and time) rather than absolute, since reports were received from health centres and hospitals only and cover a period of expansion in access to care as well as variation in diagnostic capacity and/or reporting accuracy over time. The data can be used to stratify areas for improved targeting of control efforts to steadily reduce incidence. Geographical units (zones or similar defined areas) reported incidence of confirmed malaria ranging from 0.2/1,000 per year to 73.2 cases per 1,000 per year, with 26 of 86 units averaging less than five confirmed cases per 1,000 persons per year. The incidence estimates for all malaria indicators also provide a baseline against which to gauge future progress towards elimination. Alignment of control planning and reporting with disease reporting units would facilitate assessment of control effectiveness. Inclusion of climate information over this time period and extension of the dataset to more years is needed to clarify the impact of control measures compared to natural cycles on the observed changes.

## Abbreviations

CDC: Centers for disease control and prevention; EC: Ethiopian calendar; FMOH: Federal Ministry of Health; GC: Gregorian calendar; HMIS: Health Management Information System; IDSR: Integrated disease surveillance and response; IRS: Indoor residual spraying; LLIN: Long-lasting insecticidal net; PHEM: Public Health Emergency Management; SNNPR: Southern Nations Nationalities and Peoples’ Region; WHO: World Health Organization.

## Competing interests

The authors declare that they have no competing interests.

## Authors’ contributions

DJ, MW, AAl, AT, NA, and AAd set up and managed the IDSR database; DJ, WD, ZT, TG, FOR and PG devised data management and analysis plan and did data analysis; AWM did the mapping; PG and DJ wrote the draft manuscript with extensive input from TG, FOR, AT, MW, AAl and NA; all authors commented on the draft manuscript and approved the final version.

## Supplementary Material

Additional file 1 **IDSR reporting form, 2004 to 2009.** Scanned copy of IDSR monthly form used during 2004 to 2009 at health centres and hospitals showing data items reported. Click here for file

Additional file 2 **Malaria indicators (original and generated) in the IDSR database.** List of variables relating to malaria in the IDSR database, name changes and variable definitions for old and newly created variables. Click here for file

Additional file 3 **Regions, Zones and Populations of IDSR reporting units, 2007.** Lists of 108 reporting units in 2008/2009 showing their regions and names, names as spelled in census, name of original zone in 2004/2005, populations of each unit from Census 2007 and how the 108 reporting units were assigned in collapsing of new to old units. Click here for file

Additional file 4 **Reporting periods and number of eligible months for 108 IDSR reporting units.** List of 108 eligible units, the starting and ending year and month of eligibility for reporting and the number of eligible months. Click here for file

Additional file 5 **Reporting completeness by month, site and overall for 108 reporting units.** List of 108 reporting units by region; the number of eligible months and the number and % of months actually reported; the number of eligible sites and the number and % actually stated to be reporting. Click here for file

Additional file 6 **Average annual incidence of reported total (clinical and confirmed) out-patient malaria per 1000 persons by zone, Ethiopia 2004–2009.** List of 86 collapsed reporting units by region, with census 2007 population, annual incidence/1000 of total out-patient malaria cases each year from 2004/2005 to 2008/2009, average annual incidence per year for 2004 to 2009, and expected annual number of total malaria cases. The reporting units with >50,000 expected total malaria cases per year are highlighted. Click here for file

Additional file 7 **Average annual incidence of confirmed outpatient malaria per 1000 persons by zone, Ethiopia 2004–2009.** List of 86 collapsed reporting units by region, with census 2007 population, annual incidence/1000 of confirmed out-patient malaria cases each year from 2004/2005 to 2008/2009, average annual incidence per year for 2004 to 2009, and expected annual number of confirmed cases. The reporting units with >15,000 expected confirmed malaria cases per year are highlighted. Click here for file
