# Supplementary material for: Analysis of malaria surveillance data in Ethiopia: what can be learned from the Integrated Disease Surveillance and Response System?
Source: Malar J. 2012 Sep 17;11:330. doi: 10.1186/1475-2875-11-330 (PMC3528460; doi:10.1186/1475-2875-11-330)
Supplement: Additional file 1 — IDSR reporting form, 2004 to 2009. Scanned copy of IDSR monthly form used during 2004 to 2009 at health centres and hospitals showing data items reported. [file 1475-2875-11-330-S1.doc]

Additional file 1: IDSR reporting form, 2004 to 2009
