# Supplementary material for: Analysis of malaria surveillance data in Ethiopia: what can be learned from the Integrated Disease Surveillance and Response System?
Source: Malar J. 2012 Sep 17;11:330. doi: 10.1186/1475-2875-11-330 (PMC3528460; doi:10.1186/1475-2875-11-330)
Supplement: Additional file 2 — Malaria indicators (original and generated) in the IDSR database. List of variables relating to malaria in the IDSR database, name changes and variable definitions for old and newly created variables. [file 1475-2875-11-330-S2.doc]

Additional file 2: Malaria indicators (original and generated) in the IDSR database

| Variable Number | Old name in EpiInfo IDSR database | New name in Access IDSR Malaria Database | Name Change | Notes |
| --- | --- | --- | --- | --- |
| 1 | Province | Region | Yes |  |
| 2 | District | Zone | Yes |  |
| 3 | Year | Year | No | GC year |
| 4 | MonthNumber | MonthNumber | No | GC Calendar Month |
| 5 | NumReportingSites | NumReportingSites | No | Eligible sites (health centres or hospitals) |
| 6 | NumSitesReportedTimely | NumSitesReportedTimely | No | Sites reporting on time |
| 7 | NumSitesReportedLate | NumSitesReportedLate | No | Sites reporting late |
| 8 |  | NumSitesReportedTimelyOrLate | New | Sites reporting (Sum of 6 and 7) |
| 9 |  | MalTotOutPt | New | TOTAL OUTPATIENT MALARIA CASES, ALL AGES  (sum of 14 and 17) |
| 10 |  | MalTotInPt | New | TOTAL INPATIENT MALARIA CASES, ALL AGES  (sum of 15 and 18) |
| 11 |  | MalTotInPtDth | New | TOTAL INPATIENT MALARIA DEATHS, ALL AGES  (sum of 16 and 19) |
| 12 |  | MalPfOutPt | New | TOTAL CONFIRMED *P.falciparum* OUTPATIENT CASES, ALL AGES  (sum of 27 and 29) |
| 13 |  | MalPvOutPt | New | TOTAL CONFIRMED *P.vivax* OUTPATIENT CASES , ALL AGES  (sum of 28 and 30) |
| 14 | MalL5yOutPtCases | MalL5yOutPtCases | No | OUTPATIENT MALARIA CASES IN < 5 year olds |
| 15 | MalL5yInptCases | MalL5yInPtCases | Yes | INPATIENT MALARIA CASES IN < 5 year olds |
| 16 | MalL5yInptDeaths | MalL5yInPtDeaths | Yes | MALARIA DEATHS IN < 5 year olds |
| 17 | MalGE5yOutPtCases | MalGE5yOutPtCases | No | OUTPATIENT MALARIA CASES IN 5 yrs and above |
| 18 | MalGE5yInPtCases | MalGE5yInPtCases | No | INPATIENT MALARIA CASES IN 5 yrs and above |
| 19 | MalGE5yInPtDeaths | MalGE5yInPtDeaths | No | MALARIA DEATHS IN 5 yrs and above |
| 20 | MalL5ySevAnemiaInPtCases | MalL5ySevAnmInPtCases | Yes | MALARIA WITH SEVERE ANEMIA INPATIENTS IN <5 YEAR OLDS |
| 21 | MalL5ySevAnemiaInptDeaths | MalL5ySevAnmInPtDeaths | Yes | MALARIA WITH SEVERE ANEMIA DEATHS IN <5 YEAR OLDS |
| 22 | MalG5SevAnemiaInptCases | MalGE5ySevAnmInPtCases | Yes | MALARIA WITH SEVERE ANEMIA INPATIENTS IN 5 YEAR OLDS AND ABOVE |
| 23 | MalG5yWithSevAnemiaInptDeaths | MalGE5ySevAnmInPtDeaths | Yes | MALARIA WITH SEVERE ANEMIA DEATHS IN 5 YEAR OLDS AND ABOVE |
| 24 | MalariaInPregnancyOutPtCases | MalPregOutPtCases | Yes | MALARIA IN PREGNANCY OUTPATIENTS |
| 25 | MalariaInPregnancyInptCases | MalPregInPtCases | Yes | MALARIA IN PREGNANCY INPATIENTS |
| 26 | MalariaInPregnancyInptDeaths | MalPregInPtDeaths | Yes | MALARIA IN PREGNANCY DEATHS |
| 27 | MalL5yLabOutPtCases | MalL5yConfPfOutPtCases | Yes | CONFIRMED *P.falciparum* MALARIA OUTPATIENTS IN <5 YEAR OLDS (Assume Pf cases only) |
| 28 | UncomplicatedMalPVivax | MalL5yConfPvOutPtCases | Yes | CONFIRMED *P.vivax* MALARIA OUTPATIENTS IN <5 YEAR OLDS (Assume Less than 5y) |
| 29 | MalGE5yLabOutPtCases | MalGE5yConfPfOutPtCases | Yes | CONFIRMED *P.falciparum* MALARIA OUTPATIENTS IN 5 YEAR OLDS AND ABOVE (Assume Pf cases only) |
| 30 | UncomplicatedMalG5LabConfPVivax | MalGE5yConfPvOutPtCases | Yes | CONFIRMED *P.vivax* MALARIA OUTPATIENTS IN 5 YEAR OLDS AND ABOVE |
