# Supplementary material for: Analysis of malaria surveillance data in Ethiopia: what can be learned from the Integrated Disease Surveillance and Response System?
Source: Malar J. 2012 Sep 17;11:330. doi: 10.1186/1475-2875-11-330 (PMC3528460; doi:10.1186/1475-2875-11-330)
Supplement: Additional file 3 — Regions, Zones and Populations of IDSR reporting units, 2007. Lists of 108 reporting units in 2008/2009 showing their regions and names, names as spelled in census, name of original zone in 2004/2005, populations of each unit from Census 2007 and how the 108 reporting units were assigned in collapsing of new to old units. [file 1475-2875-11-330-S3.doc]

Additional file 3: Regions, Zones and Populations of IDSR reporting units, 2007

Notes on Additional file 3

Zone: Name of reporting unit in IDSR (zone or referral hospital)

Census name: Name of zone or area in 2007 census

Zone2: Names of ‘collapsed’ zones for continuity of reporting; previous name of ‘split zones’ or recently added town reporting units

Pop2007: 2007 census population for original Zone if available

Pop2007_2: 2007 census population for collapsed zones: adjusted to include recently added town units or to join together recently split zones.
