# Supplementary material for: Analysis of malaria surveillance data in Ethiopia: what can be learned from the Integrated Disease Surveillance and Response System?
Source: Malar J. 2012 Sep 17;11:330. doi: 10.1186/1475-2875-11-330 (PMC3528460; doi:10.1186/1475-2875-11-330)
Supplement: Additional file 4 — Reporting periods and number of eligible months for 108 IDSR reporting units. List of 108 eligible units, the starting and ending year and month of eligibility for reporting and the number of eligible months. [file 1475-2875-11-330-S4.doc]

Additional file 4: Reporting periods and number of eligible months for 108 IDSR reporting units
