# Supplementary material for: Analysis of malaria surveillance data in Ethiopia: what can be learned from the Integrated Disease Surveillance and Response System?
Source: Malar J. 2012 Sep 17;11:330. doi: 10.1186/1475-2875-11-330 (PMC3528460; doi:10.1186/1475-2875-11-330)
Supplement: Additional file 5 — Reporting completeness by month, site and overall for 108 reporting units. List of 108 reporting units by region; the number of eligible months and the number and % of months actually reported; the number of eligible sites and the number and % actually stated to be reporting. [file 1475-2875-11-330-S5.doc]

Additional file 5**:** Reporting completeness by month, site and overall for 108 reporting units.
