# Supplementary material for: Analysis of malaria surveillance data in Ethiopia: what can be learned from the Integrated Disease Surveillance and Response System?
Source: Malar J. 2012 Sep 17;11:330. doi: 10.1186/1475-2875-11-330 (PMC3528460; doi:10.1186/1475-2875-11-330)
Supplement: Additional file 7 — Average annual incidence of confirmed outpatient malaria per 1000 persons by zone, Ethiopia 2004–2009. List of 86 collapsed reporting units by region, with census 2007 population, annual incidence/1000 of confirmed out-patient malaria cases each year from 2004/2005 to 2008/2009, average annual incidence per year for 2004 to 2009, and expected annual number of confirmed cases. The reporting units with >15,000 expected confirmed malaria cases per year are highlighted. [file 1475-2875-11-330-S7.doc]

Additional file 7: Average annual incidence of confirmed outpatient malaria per 1000 persons by zone, Ethiopia 2004-2009.
